# Improving Communication in the Red Meat Industry: Opinion Leaders May Be Used to Inform the Public About Farm Practices and Their Animal Welfare Implications

**DOI:** 10.3389/fpsyg.2022.876034

**Published:** 2022-08-02

**Authors:** Carolina A. Munoz, Lauren M. Hemsworth, Paul H. Hemsworth, Maxine Rice, Grahame J. Coleman

**Affiliations:** Animal Welfare Science Centre, Faculty of Veterinary and Agricultural Sciences, The University of Melbourne, Melbourne, VIC, Australia

**Keywords:** livestock welfare, general public attitudes, education, husbandry practices, trusted advisors

## Abstract

Opinion leaders (OLs) within the community may lead debate on animal welfare issues and provide a path for information to their social networks. However, little is known about OLs’ attitudes, activities conducted to express their views about animal welfare and whether they are well informed, or not, about husbandry practices in the red meat industry. This study aimed to (1) identify OLs in the general public and among producers and (2) compare OLs and non-OLs’ attitudes, knowledge and actions to express their views about the red meat industry. Two questionnaires, one for the Australian general public (*n* = 501) and one for Australian red meat producers (*n* = 200), were developed to identify general attitudes. From these questionnaires, OLs were identified using a two-step cluster analysis. Subsequently, a sub-sample of 19 OLs (including the public and producers) participated in a follow-up phone interview. Results disclosed some clear OLs’ characteristics. Public OLs held more negative perceptions of the red meat industry and perceived they had more knowledge about husbandry procedures. However, their actual knowledge about animal husbandry was not different from non-OLs. Public OLs also used and trusted social and internet media more than did non-OLs. In the producer group, a large percentage of OLs were identified (64.0% compared to 29.1% in the public group). Producer OLs had more actual knowledge about animal husbandry and engaged in more behaviours to express dissatisfaction with the industry than non-OLs (dissatisfaction in relation to the image of the red meat industry). Unlike the public respondents, this group used conventional media more than social and internet media, and their levels of trust in all kinds of media were low. While there were clear differences, both groups believed that is important to increase communication and educate about farm practices. This may present an opportunity to develop an opinion leader intervention strategy where informed OLs could later disseminate accurate information to their social networks. Further studies should test if sustained and facilitated educational sessions between public and producer OLs can assist in increasing communication, knowledge and perhaps, may assist in achieving convergence of concerns and expectations between both groups.

## Introduction

With urbanization, there has been a clear disconnect between the urban population and livestock producers. People now have less connection to rural life and less understanding of how their food is produced than in the past. In Australia, a recent report disclosed that 40% of survey respondents (total survey respondents was 1,521) felt they did not have enough information to understand what happens in the agricultural industry and 42% of respondents felt there is too much and often conflicting information about animal welfare (Futureye, 2018). Research indicates the need to increase knowledge of farm practices and animal welfare within the general public, but there is also the need to increase communication pathways between producers and the public. This would enable, both parties to share their concerns and expectations, and reduce the gaps between the public and livestock producers.

To increase communication and obtain a better shared understanding between producers and the general public, it may be possible to use opinion leaders and their social networks. Opinion leaders are generally more engaged individuals, characterised by the high interest and engagement with news and high tendency to share content (Barberá et al., 2015; Dubois et al., 2020). Opinion leaders are not necessarily the ones that propose new ideas or are the earliest adopters of innovations. However, they tend to investigate and monitor what is happening with certain topics and use their influence when they perceive that the advantages of certain ideas/innovations are evident (Valente and Pumpuang, 2007). For the purpose of this study, opinion leaders were identified on the basis that they reported being used as sources of information about farm animal welfare and provided such information to the people that they encountered. According to the two-step flow theory (Katz and Lazarsfeld, 1955), which was latter modified to a multi-step flow theory (Weimann, 1982), information flows from a range of sources (e.g., television, newspapers, and social media) to opinion leaders in the community, who then disseminate it to less engaged individuals. The use of opinion leaders, as facilitators of targeted information, may be a cost-effective strategy, that is adaptable, and can reach many people in a short amount of time. Some argued that the role of opinion leaders may be diminished by the prevalent use of direct messaging due to a more fragmented society (Bennett and Manheim, 2006). Others, however, proposed that social media provides new opportunities for opinion leaders, as they can share their views and information to a wider audience (Messing and Westwood, 2014; Turcotte et al., 2015; Bode, 2016).

Intervention strategies that include opinion leaders, such as The Popular Opinion Leader Intervention Model, have been largely used in the medical field to drive positive behavioural change in the community (Kelly et al., 1992; National Institute of Mental Health and Collaborative HIV/STD Prevention Trial Group, 2010; Theall et al., 2015). As an example, Theall et al. (2015) recruited and trained 65 opinion leaders to diffuse intervention messages related to HIV-risk behaviour. Opinion leaders-followers were surveyed 1 year later, and the results showed significant behavioural and knowledge changes. Average sexual risk score declined from 15.3 to 11.9 (*p* < 0.001) and the number of HIV knowledge score (based on % correct) increased from 67.2 to 76.8% (*p* < 0.001). In other fields, it has been shown that opinion leaders can influence others into adopting pro-environmental behaviours when their advice is sought (Geiger et al., 2019).

In the livestock industry sector, there has been limited research on opinion leaders. A study conducted by Coleman et al. (2017) identified that opinion leaders within the general public held more positive attitudes to farm animal rights and to the importance of animal welfare and more negative attitudes towards the livestock industries. Opinion leaders were also differentiated from non-opinion leaders by their perceived, but not actual, knowledge of the livestock industries. The only demographic variable that distinguished this group was age, with younger people more likely to consider themselves opinion leaders. Because opinion leaders identified by Coleman et al. (2017) held more negative attitudes towards the livestock industries and perceived they had more knowledge than what they actually had, they may present a risk to the livestock industries (Coleman, 2018). To the extent that opinion leaders exert community influence, they may increase pressure on the general public, producers, governments, and regulators to change practices.

Some studies have investigated the influence of the media in shaping consumers’ attitudes and behaviour. A few studies have reported some impact on meat demand, particularly pork and chicken, after exposure to welfare information provided by media sources (Tonsor and Olynk, 2011). Similarly, increased community discussion and an increase in the perceived importance of conditions for live sheep transport has been reported after a media campaign exposing animal cruelty (Rice et al., 2020). Possibly, opinion leaders could influence (exacerbate or mitigate) the general public responses to the information provided by social or conventional media about animal welfare issues in the red meat industry. However, little research has been done to identify opinion leaders’ attitudes, knowledge and activities conducted to express their views in relation to the red meat industry and whether they are well informed, or not, about the industry and farm practices. To the authors’ knowledge, there are no previous studies identifying opinion leaders among producers and their attitudes, knowledge, and activities to engage with the general public. To address these knowledge gaps, this present study aimed to (1) identify opinion leaders in the Australian general public and among Australian red meat producers and (2) compare opinion leaders and non-opinion leaders’ attitudes, knowledge and actions/activities to express their views about the red meat industry. By understanding the key characteristics of opinion leaders and similarities and differences, it may be possible to identify strategies that allow better communication and education about farm animal welfare in the red meat industry.

## Materials and Methods

Two main data sets were used to identify opinion leaders’ attitudes, knowledge and behaviour. (1) A national questionnaire (quantitative data), comprising *n* = 501 responses from the general public and *n* = 200 from sheep and beef producers and (2) One-on-one phone interviews with opinion leaders (*n* = 19, qualitative data). Further details of the methodology are provided below.

### National Questionnaires

Two questionnaires, one for the general public and one for producers, were developed to identify general attitudes towards the red meat industry. The questionnaires were developed using an iterative process beginning with questionnaires that had been developed by the Animal Welfare Science Centre (AWSC) for a range of livestock industries including the pork, egg and red meat industries. The draft version of the questionnaires was reviewed by key stakeholders from the sheep industry, beef cattle industry, Meat and Livestock Australia, The Royal Society for the Prevention of Cruelty to Animals (RSPCA) and the AWSC. The questionnaires were then piloted to gather further feedback. The final version of the questionnaires comprised five sections.

1. Demographics (and farm demographics in producer questionnaire)
2. Animal welfare
3. Knowledge of farm animals and farm animal welfare
4. Attitudes towards red meat farming practices
5. Behaviour in relation to farm animal welfare

Sections 3, 4, and 5 of the questionnaires are the most relevant to this study (refer to Hemsworth et al., 2021 for further details). Section 3, knowledge of farm animals and farm animal welfare, included questions in relation to perceived knowledge such as *How much do you feel you know about beef cattle and sheep production in Australia?* Perceived knowledge was scored using a scale from 1 (nothing at all) to 5 (a lot). Questions about actual knowledge comprised multiple choice questions about farm practices such as *What do the following farming practices (e.g., dehorning, castration, mulesing, etc.) involve?* Actual knowledge was calculated based on percentage of correct answers. Section 4, Attitudes towards red meat farming practices, included Likert-scale questions such as *To what extent do you approve or disapprove of the following procedures/practices carried out on beef cattle and/or sheep (e.g., mulesing, castration, dehorning, etc.)?* The theory of planned behaviour was used to develop the attitudinal statements. Section 5, Behaviour in relation to farm animal welfare, included Likert-scale questions such as *Have you done any of the following activities to express your dissatisfaction with any aspect of sheep and beef cattle farming (e.g., shared information on social media, called a radio talk back segment, attended a public rally, etc.)?*

To deliver the questionnaires to the general public (*n* = 501), a specialised market and social research data collection agency (I-View Pty Ltd., Melbourne, Australia) was contracted. Random telephone recruitment (CATI) was used (for details of this methodology, refer to Hemsworth et al., 2021). To compensate for a tendency for female responders to predominate, equal numbers of males and females were contacted. To deliver the questionnaire to producers (*n* = 200), a CATI questionnaire was conducted by an agricultural market research company (Kg2, Sydney, Australia) using contacts drawn from a proprietary contact list. All data collection commenced on 21 March 2018 and was completed on 16 April 2018. The average duration of the telephone interviews was about 30–40 min.

### One-on-One Interviews With Opinion Leaders

From the national questionnaires, opinion leaders in the general public sample and the producer sample were identified and invited to participate in a follow-up phone interview. The method for opinion leaders’ identification is described below in the Results section “Identification of Opinion Leaders.” A total of 19 opinion leaders (*n* = 10 general public and *n* = 9 producers) were recruited. The phone interviews aimed to obtain further insights into the motivations behind opinion leaders’ views of the red meat industry. Thus, the questions included in the phone interviews were developed based on the national questionnaire data (see details below). Responses to these questions were probed further if the interviewer needed to seek clarification of the responses. The average duration of the interviews was 40–60 min, and they were all conducted by the same researcher (CM).

#### Questions Asked to General Public Opinion Leaders

What is your opinion of the red meat industry, and why?
Do you take actions in opposition to the red meat industry? what actions? why?
Why are you motivated to take these actions?
What outcomes do you want to achieve?
What changes would you like to see that would change your view on the red meat industry?

#### Questions Asked to Producer Opinion Leaders

Do you actively promote the red meat industry? Why? If yes, how?
What do you think the public thinks about animal welfare in red meat production? (Any difference between sheep and beef cattle?)
Do you think there is enough communication between producers and the general public on general practices in livestock production?
What do you think could be done to improve public perception, to allow greater convergence in views between producers and public?

### Statistical Analysis

Questionnaire data were analysed using Principal Components Analysis (PCA) followed by either a Varimax or an Oblimin rotation to identify commonalities amongst the questionnaire items. Questionnaire items that were established as belonging to a common underlying component were summed to produce a composite score (scale score) for that component. The scale definitions are summarised in Table 1. From the questionnaire data, opinion leaders were identified using a two-step cluster analysis of their responses to three questions adapted from Childers (1986). The questions used to identify opinion leaders were (1) *During the past 6 months, how many people have you told about farm animal welfare?* (2) *Compared with your friends, how likely are you to be asked about farm animal welfare?* and (3) *In all of your discussions with friends and neighbours how often are you used as a source of advice on farm animal welfare?* ANOVAS, *t*-tests, and Chi-square analyses were then conducted to investigate differences in meat consumption, age and education levels, knowledge and attitudes between opinion leaders and non-opinion leaders.

**Table 1:** Attitude scale definitions.

Component label	Description
Red meat attributes	Questions related to healthiness of red meat
Red meat animal rights	Beliefs that sheep and beef cattle have the same feelings and the same rights as other domestic animals
Trust livestock people	Trust animal workers to care for animals well
Approval of husbandry practices	Approve of crutching, branding, mulesing, etc.
Use of medication on animals	Receive appropriate vaccinations, medications
Land beef transport conditions	Need for good cattle land transport conditions
Sea beef cattle transport conditions	Need for good cattle sea transport conditions
Land sheep transport conditions	Need for good sheep land transport conditions
Sea sheep transport conditions	Need for good sheep sea transport conditions
Public engagement beliefs	Need to actively promote sheep and cattle welfare
Negative normative beliefs	Friends and relatives would expect people to actively oppose
Positive normative beliefs	Friends and relatives would expect people to actively support
Difficult to act	Difficult to engage in community actions
Easy to act	Easy to engage in community actions
General welfare	Social contact, protection, exercise, outdoor access, etc.
Animal welfare humane	Animal welfare involves humane animal care/treatment
Animal welfare handling	Animal welfare involves appropriate animal handling
Animal welfare people animals	Animal welfare involves a positive human-animal relationship
Commercial media	Trust commercial media for information
Social and internet media	Use Social and internet media for information
Conventional media	Use conventional media for information
Trust social and internet media	Trust Social and internet media for information
Trust conventional media	Trust conventional media for information
Community behaviour	Actions taken in favour or against the industry

Questions in the national questionnaire were grouped into common themes (referred to as components) using Principal Component Analysis. The description above relates to the common themes identified in the questions that were included in each component.

Interviews on opinion leaders were recorded, transcribed, and coded using NVivo10 qualitative data analysis software (QSR International Pty Ltd.). Thematic analysis was used to analyse the transcripts. Analysis was conducted using a grounded theory approach; thus, codes were identified as they arose from the data (Glaser and Strauss, 1967).

## Results

### Identification of Opinion Leaders

#### The General Public

Three questions adapted from Childers (1986) were used in a two-step cluster analysis to identify those respondents who were used within their social group as a local source of information regarding farm animal welfare (see Table 2). The order of cases was first sorted into random order. Two groups were identified, those who showed a high level of activity as measured by these questions, and those who did not. The cluster analysis of the responses from the general public identified 146 opinion leaders (29.1%) and the remaining 355 respondents were in the non-opinion leader group. Cluster means for the three items are given in Table 2. The Silhouette coefficient for these clusters was 0.6 which reflects a good fit (Kaufman and Rousseeuw, 1990).

**Table 2:** Group means for the two clusters identified among the general public and the red meat producers, opinion leaders and non-opinion leaders, standard deviations are provided in brackets.

Questionnaire items	General public		Producers	
	Opinion-leaders	Non-opinion leaders	Opinion-leaders	Non-opinion leaders
	(*n* = 146)	(*n* = 355)	(*n* = 128)	(*n* = 72)
During the past 6 months, how many people have you told about farm animal welfare?	3.87 (1.14)	1.70 (1.01)	4.54 (0.94)	2.19 (1.26)
Compared with your friends, how likely are you to be asked about farm animal welfare?	3.74 (1.00)	1.64 (0.87)	4.05 (1.01)	2.06 (1.05)
In all of your discussions with friends and neighbours how often are you used as a source of advice on farm animal welfare?	3.15 (0.94)	1.35 (0.59)	3.66 (0.88)	1.88 (0.87)

#### Red Meat Producers

Based on the same three questionnaire items, 128 opinion leaders (64.0%) were identified within the producer respondents, and the remaining 72 respondents were in the non-opinion leader group. The Silhouette coefficient for these clusters was also 0.6. The proportion of producers that were identified as opinion leaders was significantly higher than for the public sample (29.1% of the public sample vs. 64.0% of the producer sample).

### Comparison Between Opinion Leaders and Non-opinion Leaders

#### Meat Consumption, Gender, Age, and Education Level

Amongst the general public respondents, while there was a tendency for more opinion leaders to be vegetarian or vegan (17.1% opinion leaders and 7.9% non-opinion leaders), the majority in both groups were meat-eaters (82.9% opinion leaders and 92.1% non-opinion leaders). Overall, females predominated amongst the opinion leaders of the general public respondents compared to non-opinion leaders (65.1% vs. 49.3; *χ*^2^_1_ = 10.36, *p* < 0.01%), however, there were no significant differences between public opinion-leaders and non-opinion leaders in age distribution (*χ*^2^_5_ = 8.56, *p* = 0.13) or education level (*χ*^2^_5_ = 9.43, *p* = 0.09). Producers all described themselves as “meat and vegetable eaters.” For this group, there were no significant differences between opinion leaders and non-opinion leaders in gender (*χ*^2^_5_ = 1.04, *p* = 0.31), age distribution (*χ*^2^_5_ = 7.26, *p* = 0.20), or education level (*χ*^2^_5_ = 4.12, *p* = 0.52).

#### Perceived and Actual Knowledge About the Red Meat Industry

Amongst the general public, questionnaire respondents perceived that their knowledge about the red meat industry was moderate (sheep: mean 3.06; beef: mean 3.19 on a 5-point scale). Overall, the actual knowledge of the respondents (in terms of percentage of correct answers) ranged from 15.38 to 100% with an average score of 72.34% correct answers. Opinion leaders in the general public perceived that their knowledge about beef cattle and sheep production was higher than did non-opinion leaders (beef: 3.63 vs. 3.01, *t*_499_ = 5.69, *p* < 0.01; sheep: 3.51 vs. 2.87, *t*_499_ = 5.71, *p* < 0.01). However, when actual knowledge (knowledge score, that is, percentage of correct answers) was compared between opinion leaders and non-opinion leaders, there was no difference (72.18% vs. 72.41%). In the producer group, *n* = 81 respondents reported to be beef producers, *n* = 52 sheep producers, and *n* = 65 were both sheep and cattle producers. Knowledge of husbandry practices ranged from 46.15% to 100% correct answers, with an average of 93.27% and the majority scoring 100%. Not surprisingly, this is considerably higher than for the public sample. Producer opinion leaders perceived that their knowledge about beef cattle husbandry practices was not different from non-opinion leaders (4.31 vs. 4.13, *t*_198_ = 1.45, *p* = 0.15) but perceived that their knowledge about sheep husbandry practices was higher than that of non-opinion leaders (4.13 vs. 3.65, *t*_198_ = 2.82, *p* < 0.01). When actual knowledge (knowledge score) was compared between producer opinion leaders and non-opinion leaders, opinion leaders’ knowledge about sheep and cattle husbandry practices was higher than that of non-opinion leaders (95.01 vs. 90.17; *t*_198_ = 3.64, *p* < 0.01).

#### General Attitudes Towards the Red Meat Industry

Table 3 presents the attitude results comparing opinion leaders and non-opinion leaders for the general public sample. In general, both groups, opinion leaders and non-opinion leaders, tended to hold somewhat positive views towards the red meat industry. For example, questionnaire statements in relation to trust in the red meat industry and red meat attributes were all scored 3+ out of a maximum score of 5. However, comparisons between the two samples showed that opinion leaders tended to hold more negative views of the red meat industries. For instance, opinion leaders considered red meat less healthy (referred in the table as red meat attributes), had lower approval levels for husbandry practices (referred as approval of husbandry practices), rated sheep and cattle rights as more similar to domestic animals (referred as red meat animal rights) and had lower approval levels for sheep and cattle transport conditions (referred as sea beef transport conditions, land sheep transport conditions, and sea sheep transport conditions). In addition, opinion leaders in the general public sample more strongly believed in the need to actively promote sheep and cattle welfare (referred as public engagement beliefs), more strongly believed that friends and relatives would expect them to actively support animal welfare (referred as positive normative beliefs) and that it was easy to engage in community actions (referred as easy to act). They also were more likely to use all kinds of media for information, but significantly used and trusted social and internet media more than non-opinion leaders.

**Table 3:** Comparisons between the general public opinion leaders and non-opinion leaders on attitudes towards red meat farming practices and community behaviour (df = 499).

PCA components^*^	Opinion leaders	Non-opinion leaders	*t*	Sig	Cohen’s D
	Mean score	Mean score			
Red meat attributes	**3.35**	**3.76**	**−4.25**	**0.00**	**−0.38**
Red meat animal rights	**4.17**	**3.90**	**2.66**	**0.01**	**0.24**
Trust in the red meat industry	**3.06**	**3.49**	**−3.86**	**0.00**	**−0.35**
Approval of husbandry practices	2.95	3.08	−1.46	0.15	−0.13
Use of medication on animals	4.51	4.57	−0.83	0.41	−0.07
Land beef transport conditions	**2.29**	**2.57**	**−2.61**	**0.01**	**−0.23**
Sea beef transport conditions	**1.79**	**2.23**	**−4.23**	**0.00**	**−0.38**
Land sheep transport conditions	**2.07**	**2.46**	**−3.52**	**0.00**	**−0.32**
Sea sheep transport conditions	**1.68**	**2.17**	**−4.77**	**0.00**	**−0.43**
Public engagement beliefs	**4.00**	**3.31**	**6.46**	**0.00**	**0.58**
Negative normative beliefs	**2.45**	**3.07**	**−5.66**	**0.00**	**−0.51**
Positive normative beliefs	**3.67**	**3.14**	**5.11**	**0.00**	**0.46**
Difficult to act	**2.57**	**2.92**	**−3.47**	**0.00**	**−0.31**
Easy to act	**3.76**	**2.86**	**8.40**	**0.00**	**0.75**
General welfare	4.81	4.75	1.50	0.13	0.13
Animal welfare humane	4.62	4.48	1.60	0.11	0.14
Animal welfare handling	4.27	4.29	−0.20	0.84	−0.02
Animal welfare people animals	**4.20**	**4.01**	**2.08**	**0.04**	**0.19**
Commercial media	**2.20**	**1.94**	**3.84**	**0.00**	**0.34**
Social and internet media	**3.30**	**2.51**	**8.90**	**0.00**	**0.80**
Conventional media	**2.82**	**2.51**	**3.44**	**0.00**	**0.31**
Trust social and internet media	**3.16**	**2.92**	**3.35**	**0.00**	**0.30**
Trust conventional media	2.58	2.61	−0.38	0.71	−0.03
Community behaviour	**3.46**	**1.98**	**8.27**	**0.00**	**0.74**

* Label definitions – see *Table 1* for details. Values highlighted in the table are statistically significant.

Table 4 presents attitude results comparing opinion leaders and non-opinion leaders for the red meat producer sample. Comparisons between the two groups, producer opinion leaders and non-opinion leaders, showed that both groups tended to share similar views of the red meat industry and animal welfare. They tended to hold positive views about the industry and more negative views and low trust on commercial and social media. Main differences between opinion leaders and non-opinion leaders were in relation to normative beliefs and community behaviour. That is, opinion leaders in the producer group more strongly believed that friends and relatives would expect them to actively support animal welfare (referred in the table as positive normative beliefs) and that it was easy to engage in community actions (referred as easy to act). This agrees with the public opinion leader sample. However, unlike the public respondents, opinion leaders in the producer group used conventional media more (score 3.37) than social and internet media (score 2.46) or commercial media (score 2.4). Levels of trust also differed from the public group as levels of trust in all kinds of media were relatively low, ranging from 2.39 to 2.70 out of 5.

**Table 4:** Comparisons between producer opinion leaders and non-opinion leaders on attitudes towards red meat farming practices and community behaviour (df = 198).

PCA components^*^	Opinion leaders	Non-opinion leaders	*t*	Sig	Cohen’s D
	Mean score	Mean score			
Red meat attributes	4.74	4.64	1.85	0.07	0.26
Red meat animal rights	3.80	3.99	−1.21	0.23	−0.17
Trust in the red meat industry	4.44	4.29	1.56	0.12	0.22
Approval of husbandry practices	4.10	4.08	0.23	0.82	0.03
Use of medication on animals	4.74	4.68	0.75	0.46	0.11
Land beef transport conditions	3.94	3.99	−0.42	0.68	−0.06
Sea beef transport conditions	3.76	3.70	0.42	0.68	0.06
Land sheep transport conditions	3.96	3.98	−0.22	0.82	−0.03
Sea sheep transport conditions	3.61	3.47	0.84	0.40	0.12
Public engagement beliefs	3.41	3.23	1.17	0.24	0.17
Negative normative beliefs	2.77	2.86	−0.65	0.51	−0.09
Positive normative beliefs	**3.67**	**3.35**	**2.05**	**0.04**	**0.29**
Difficult to act	2.47	2.71	−1.60	0.11	−0.23
Easy to act	**3.43**	**2.90**	**3.13**	**0.00**	**0.44**
General welfare	4.60	4.66	−1.13	0.26	−0.16
Animal welfare humane	4.65	4.53	1.25	0.21	0.03
Animal welfare handling	4.63	4.62	0.05	0.96	0.01
Animal welfare people animals	4.18	4.01	1.31	0.19	0.19
Commercial media	**2.40**	**2.05**	**3.35**	**0.00**	**0.47**
Social and internet media	**2.46**	**2.01**	**3.70**	**0.00**	**0.53**
Conventional media	3.37	3.19	1.32	0.19	0.19
Trust social and internet media	2.39	2.49	−0.97	0.34	−0.14
Trust conventional media	2.70	2.56	1.27	0.20	0.18
Community behaviour	**1.46**	**1.03**	**2.05**	**0.04**	**0.29**

* Label definitions – see *Table 1* for details. Values highlighted in the table are statistically significant.

#### Behaviours Performed to Express Dissatisfaction With the Red Meat Industry

Opinion leaders in the general public group reported engaging in twice as many behaviours/activities to express dissatisfaction with the red meat industry compared with non-opinion leaders (means 3.46 vs. 1.98, *t*_499_ = 8.27, *p* < 0.01). The most common behaviours/activities performed by opinion leaders were: “spoken to colleagues, family members or friends,” “donated money to animal welfare organisations,” “signed petitions,” and “posted/shared information about an issue on social media such as Facebook, Twitter, Instagram.” Overall, there were significantly more opinion leaders who reported being current members of an animal rights group (*χ*^2^_2_ = 12.04, *p* < 0.01) but in general the prevalence was low, with 15.1% of the opinion leader group and 5.6% of the non-opinion leader group reported to be members of an animal rights groups.

Similar to the public group, producer opinion leaders reported engaging in more behaviours/activities to express dissatisfaction with the red meat industry than non-opinion leaders (means 1.46 vs. 1.03, *t*_198_ = 2.05, *p* < 0.05). However, the frequency of most behaviours/activities performed was low. The only common behaviour/activity performed by opinion leaders in the producer group was “spoken to colleagues, family members or friends.” All of the other activities occurred with a prevalence of less than 15%. Overall, there were slightly more opinion leaders who reported being members of an animal rights group, but again, the prevalence was low and not significant, 4.7% of the opinion leader group and 0% of the non-opinion leader group reported to be members of an animal rights groups.

### One-on-One Interviews With Opinion Leaders

**Figure 1:**
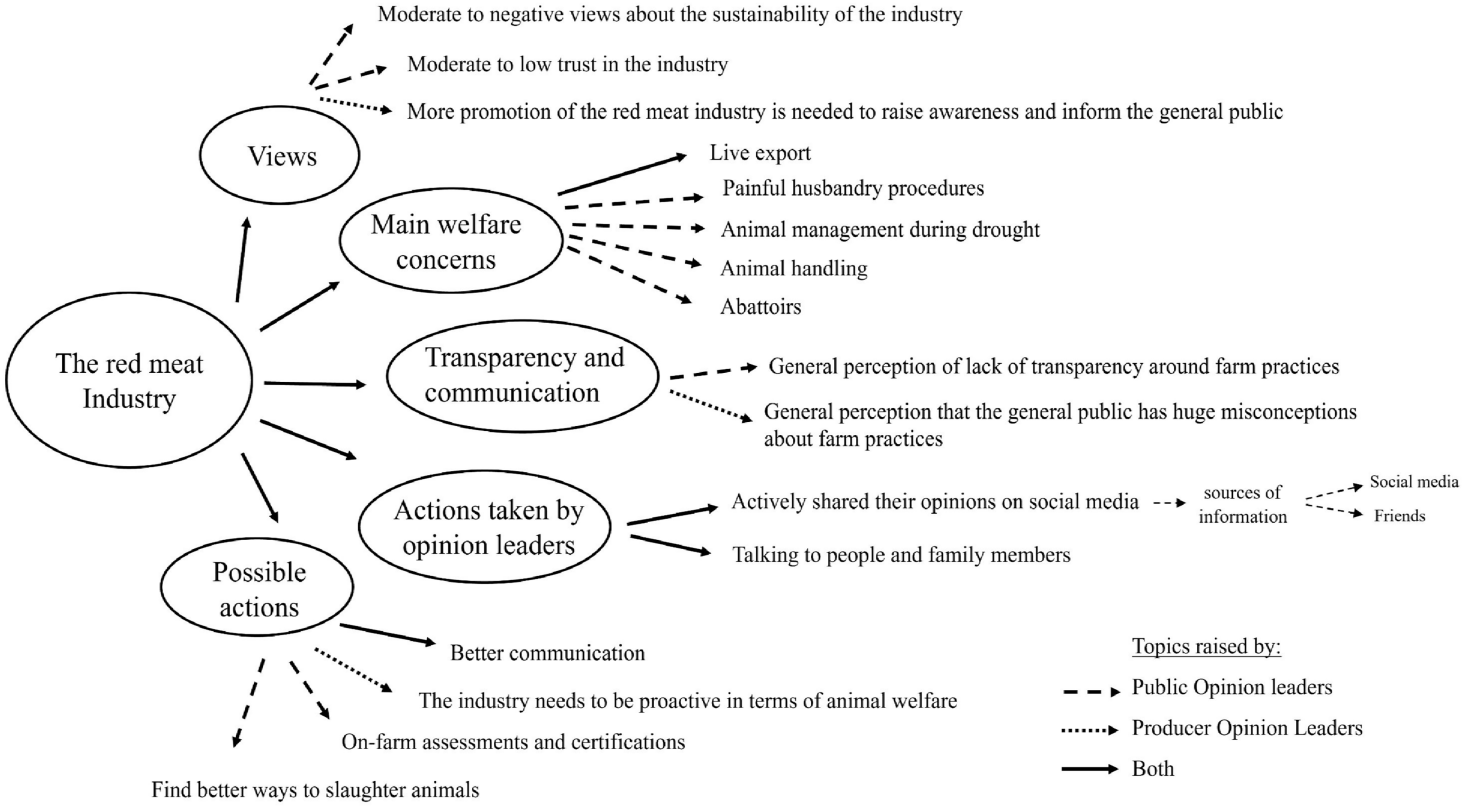
The hierarchy of themes generated using a grounded theory approach.

Following the questionnaires, a total of 19 telephone interviews were conducted, 10 interviews with the general public and nine interviews with red meat producers. For both groups, participants were recruited for interview until the researchers were satisfied with the level of data saturation (repetition) obtained from the discussions. Figure 1 illustrates the main themes identified during the one-on-one phone interviews: (1) general views of the red meat industry, (2) main welfare concerns, (3) transparency and communication, (4) actions taken by opinion leaders, and (5) possible actions to improve public perceptions towards the red meat industry.

#### General Views of the Red Meat Industry

Opinion leaders from the general public held moderate to negative views toward the red meat industry. The main reasons for their opposition to the red meat industry were related to sustainability, animal welfare concerns and ethical views. Some relevant quotes from the interviewees included “*I think the Australian industry is somewhere in the middle, probably doing better things than other countries but I’m sure there are more humane ways of doing things*,” “*I used to have a fair opinion, but recently I read about animal welfare concerns during drought, and I am not impressed*,” “*For me, sustainability is an important issue. I think Australian farmers have adopted management practices from England that suited England climate but not Australian climate*,” and “*We have used the land with no thought of what the aboriginals needed, or what the animals needed… what the land itself needs*.” Producer opinion leaders, on the other hand, had positive views towards the red meat industry and strongly believed that the public has “*huge*” misconceptions around farm practices such as poor understanding of the differences between shearing, crutching and mulesing and the importance/justification of these practices.

#### Main Welfare Concerns

Both public and producer opinion leaders expressed great concerns about the welfare of animals exported to other countries. On this topic, producer opinion leaders recognised that “*the reputation of the red meat industry has been terribly damaged by the live export industry*.” In addition to live export, other main welfare concerns for the public opinion leaders were related to painful husbandry procedures (e.g., dehorning, mulesing), the size of the production (e.g., large farm enterprises), feedlots, animal handling at transport, sale yards and abattoirs, and farming animals in dry areas (e.g., northern Australia). Producer opinion leaders perceived that consumers’ awareness about sheep and beef cattle production is increasing, but they also believed that the public’ views and opinions are mostly influenced by supermarkets. Overall, producer opinion leaders perceived that the general public is more concerned about beef cattle welfare than sheep welfare. However, most producers also recognised that they do not know what the general public think about the industry.

#### Transparency and Communication

Both public and producer opinion leaders believed that there is a lack of communication between the general public and the red meat industry. Some relevant quotes from the public opinion leaders included ‘*I am worried about the abattoir industry. It is far too secretive, and I wonder why. Is it such a bad process to kill an animal that we have to keep it as a secret? If it is bad, I would like to see a change!’* and ‘*the industry is not very transparent with consumers. For example, what are the management practices that they do?*’. Producer opinion leaders, in contrast, stated that they provide a lot of information about the welfare and management of their animals for assurance purposes, but that information is not communicated easily to consumers or the general public. Some other relevant quotes made by the producer opinion leaders included: ‘*I think some producers are not prepared to be transparent’* and ‘*the industry not only needs to be transparent but also needs to be seen as transparent’*.

#### Actions Taken by Opinion Leaders in Opposition to or Favour of the Red Meat Industry

In general, public opinion leaders engaged in a range of community behaviours because they are trying to raise awareness or drive policy change around sustainability and animal welfare issues. Most public opinion leaders believed that people should eat less meat or ensure their products are sourced from sustainable farms. Moreover, all the opinion leaders stated that they buy their meat products from local butchers and they always encourage their friends and family to do the same. Some relevant quotes included ‘*I am happy to vocalise how I feel about the industry, I am quite vocal around my friends and on social media. I try to educate people’* and ‘*I just discuss my ideas with people, I suppose that I am trying to change their opinions’*. Producer opinion leaders actively promote the industry mainly by engaging in a conversation with people. The main reasons to engage with the community were to raise awareness/inform people and to “show the other side of the coin.” They also acknowledged that they like performing those activities as it is a “social thing to do.”

#### Possible Actions to Improve Trust Towards the Red Meat Industry

Both groups of opinion leaders want to see a more proactive industry. Public opinion leaders, for example, would like to see more actions towards the sustainability of the industry and more communication pathways between the public and the red meat industry. Specific changes/actions that the public opinion leaders would support included: (1) increased on-farm monitoring of animals (e.g., development of certification schemes by a third party such as Animals Australia), (2) improvements in food labelling, (3) improvements in legislation and law enforcement (e.g., penalties to those farmers/stock people that do not follow animal welfare standards), (4) increased transparency between abattoirs and the public and (5) government support to fund the changes/actions mentioned above.

Producer opinion leaders believed the industry needs to be more proactive in communicating ‘*good stories’* to the general public, with one interviewee commenting ‘*The industry needs to send a clear message that it is a proactive industry in terms of animal welfare, instead of a reactive industry’*. Specific actions mentioned by producer opinion leaders included: (1) industry field days or expo shows could be used as opportunities to increase communication between the parties, and (2) better industry marketing, with a consistent and clear message (e.g., the industry needs to work with retailers and processors to send the same message). While some possible actions were mentioned by producers, this group also expressed concerns about consumers’ reaction to the information and the use of adequate messengers to deliver information about the industry. Some relevant quotes on this topic included ‘*the challenge is that people have to be receptive to that information’* and ‘*promoting good stories through documentaries may be possible. However, you need to get adequate people to send those messages’*.

## Discussion

This study aimed to identify opinion leaders in the general public and among producers and to compare attitudes, knowledge and actions/activities to express their views about the red meat industry. Our results showed clear differences between public and producer opinion leaders, but also some key similarities. Overall, both groups of opinion leaders believe that is important to communicate and educate about farm practices and animal welfare in the red meat industry. This motivation may present an opportunity to develop an opinion leader intervention strategy in the red meat industry. Sustained and facilitated educational sessions/interactions between public and producer opinion leaders may assist in increasing communication, knowledge, and perhaps, convergence of concerns and expectations between the public and producers.

An important consideration here is to be clear about how opinion leaders were defined in this study. The three questions adapted from Childers (1986) ask respondents about their role as sources of farm animal welfare information but do not ask for or imply attitudes to farm animal welfare. In this sense they are attitude neutral. Within the general public, opinion leaders’ attitudes differed from those of non-opinion leaders. However, within the producer group, there were no significant differences in attitudes between opinion leaders and non-opinion leaders. It is important to note that the clusters of opinion leaders that were identified in this study using two-step cluster analysis were not unique. Yet, the fit was good (silhouette values of 0.6) and there were clear differences between the clusters in attitudes and knowledge.

There were some clear characteristics of opinion leaders identified in this study, particularly in the public sample. Opinion leaders within the general public tended to hold more negative views about the industry, held higher levels of trust on social media, were mostly meat eaters, but engaged in twice as many behaviours to express concern about the red meat industry compared to non-opinion leaders. The only demographic characteristic that distinguished this group was gender, with more females identified as opinion leaders. General public opinion leaders also perceived they have higher knowledge about the industry compared to non-opinion leaders. However, their actual knowledge of animal husbandry practices was not different from the rest of the sample. These findings are in agreement with Coleman et al. (2017) who found that opinion leaders within the general public were differentiated from non-opinion leaders by their more negative attitudes towards the livestock industries and their perceived, but not actual, knowledge of the livestock industries. In the producer sample, there were fewer clear differences. Overall, producer opinion leaders and non-opinion leaders, held similar attitudes to farm animal welfare, similar positive attitudes towards the red meat industry and low levels of trust in the media, particularly conventional and social media. Producer opinion leaders reported engaging in more behaviours/activities to express dissatisfaction with the red meat industry, however, dissatisfaction amongst producers seems to relate more to concerns about how the red meat industry is perceived and a desire to improve its image.

While there were some important differences between public and producer opinion leaders, there was an important point of agreement. Both groups of opinion leaders would like to see increased transparency and communication between producers and the general public. On the one hand, opinion leaders in the general public want to educate people, raise awareness of animal welfare issues and want to drive change in the red meat industry. Yet, if the information that opinion leaders are sharing/discussing among family members or on social media is not accurate, they may present a risk to the industry. On the other hand, producer opinion leaders want to educate about farm practices and advocate for the industry. They believed the public is not well informed and that there are important misconceptions or gaps about farm practices. However, if communication pathways are not clear, or well supported by the industry, producers will not reach a wide audience. The fact that both groups of opinion leaders are motivated to communicate a message and drive awareness may present an opportunity to develop an opinion leader intervention strategy in the red meat industry.

While there were clear opposing views between opinion leaders from the general public and producers identified in this study, these polarized views do not necessarily reflect the diversity of opinions in relation to agriculture and animal welfare within a community. Studies by Matthews (1996), Coleman et al. (2016), and Coleman (2018) have discussed the diversity of public opinions and expectations about livestock and animal welfare, particularly when different systems (extensive versus intensive systems) are compared. It must be considered, however, that depending on the degree of influence that opinion leaders have in their communities, there may be a risk of them increasing polarization of attitudes between the general public and producers.

The use of opinion leaders to deliver information to their social networks is not a new concept, and it has been successfully applied in different fields to drive positive change in a community. Some examples of programs that have used opinion leaders include promotion of mammography screening (Earp et al., 2002), tobacco prevention in schools (Perry et al., 2003; Valente et al., 2003) and HIV/STD risk reduction (Kelly et al., 1992; Sikkema et al., 2000; Latkin, 2003). While there is considerable variation in how opinion leaders are defined, selected, and trained among these studies, the use of peer opinion leaders has proven to be effective. There are two key points to be considered in relation to the effectiveness of using opinion leaders. First, research has showed that people perceive information received from opinion leaders as more credible than information received directly from the media (Berkman and Gilson, 1986). Second, by sharing information, opinion leaders motivate others in their social group to seek advice or further information (Turcotte et al., 2015). Important differences between public and producer opinion leaders need to be considered when developing intervention strategies. For example, social media was one tool commonly used by opinion leaders to express their views and opinions, particularly in the general public group. The producer group, however, expressed low levels of trust in all kinds of media. Considering these results, an intervention strategy should consist of a combination of social media and face-to-face interactions. A series of online forums and targeted field days could be used to engage/train public and producer opinion leaders.

Now, there are some limitations to this study to be considered when interpreting the results, such as the recruitment of participants and the reliance on self-reported data. While the recruitment of participants was random for the general public, it was not completely random for producers. Producers were randomly selected, but they were selected from an available database. Perhaps, this influenced the large percentage of opinion leaders in the producer group compared to the general public, as the producers recruited were more likely to be involved in surveys and studies, therefore, may be considered as more “proactive” producers. Self-reported data is another common limitation with surveys. In this study, opinion leaders were identified on the basis that they reported being used as sources of information about farm animal welfare and provided such information to the people that they encountered. It is possible that some participants did identify themselves as opinion leaders but may not be perceived as opinion leaders by their social networks. A better understanding of how others rate opinion leaders and how trustworthy they are perceived by others in their social group would increase the effectiveness of an opinion leader intervention strategy.

## Conclusion

While there were clear differences between public and producer opinion leaders, the main point of agreement was that both groups expressed the need for increased communication. This suggests that it may be possible to develop an opinion leader intervention strategy to increase communication and knowledge in the red meat industry. Due to key differences in media use and trust between the public and producers, a potential strategy to increase communication may consist of a combination of social media and face-to-face interactions. Sustained and facilitated educational sessions such as online forums and targeted field days, allowing a more active exchange of knowledge and concerns between both parties, may be a first step to engage the industry with the general public. These informed/trained opinion leaders could later disseminate accurate information to their social networks using various media sources. Further studies should test the hypothesis that an opinion leader intervention strategy can increase communication, knowledge and, perhaps, assist in achieving convergence in expectations and demands between the general public and red meat producers.

## Data Availability Statement

The datasets presented in this article are not readily available because this is in concordance with human ethics requirements. Requests to access the datasets should be directed to munoz.c@unimelb.edu.au.

## Ethics Statement

The studies involving human participants were reviewed and approved by Human Research Ethics Committee at The University of Melbourne. Written informed consent for participation was not required for this study in accordance with the national legislation and the institutional requirements.

## Author Contributions

GC, PH, LH, MR, and CM: conceptualisation, methodology and investigation. CM and GC: formal analysis and writing original draft preparation. CM, MR, and GC: data curation. All authors contributed to the article and approved the submitted version.

## Funding

The authors declare that this study received funding from Meat and Livestock Australia. The funder was not involved in the study design, collection, analysis, interpretation of data, the writing of this article or the decision to submit it for publication.

## Conflict of Interest

The authors declare that the research was conducted in the absence of any commercial or financial relationships that could be construed as a potential conflict of interest.

## Publisher’s Note

All claims expressed in this article are solely those of the authors and do not necessarily represent those of their affiliated organizations, or those of the publisher, the editors and the reviewers. Any product that may be evaluated in this article, or claim that may be made by its manufacturer, is not guaranteed or endorsed by the publisher.
